# Evolutionary Patterns in Coiled-Coils

**DOI:** 10.1093/gbe/evv007

**Published:** 2015-01-10

**Authors:** Jaroslaw Surkont, Jose B. Pereira-Leal

**Affiliations:** Instituto Gulbenkian de Ciencia, Oeiras, Portugal

**Keywords:** coiled-coil, protein evolution, phylogenetic inference, homology detection, amino acid substitutions, protein structure

## Abstract

Models of protein evolution are used to describe evolutionary processes, for phylogenetic analyses and homology detection. Widely used general models of protein evolution are biased toward globular domains and lack resolution to describe evolutionary processes for other protein types. As three-dimensional structure is a major constraint to protein evolution, specific models have been proposed for other types of proteins. Here, we consider evolutionary patterns in coiled-coil forming proteins. Coiled-coils are widespread structural domains, formed by a repeated motif of seven amino acids (heptad repeat). Coiled-coil forming proteins are frequently rods and spacers, structuring both the intracellular and the extracellular spaces that often form protein interaction interfaces. We tested the hypothesis that due to their specific structure the associated evolutionary constraints differ from those of globular proteins. We showed that substitution patterns in coiled-coil regions are different than those observed in globular regions, beyond the simple heptad repeat. Based on these substitution patterns we developed a coiled-coil specific (CC) model that in the context of phylogenetic reconstruction outperforms general models in tree likelihood, often leading to different topologies. For multidomain proteins containing both a coiled-coil region and a globular domain, we showed that a combination of the CC model and a general one gives higher likelihoods than a single model. Finally, we showed that the model can be used for homology detection to increase search sensitivity for coiled-coil proteins. The CC model, software, and other supplementary materials are available at http://www.evocell.org/cgl/resources (last accessed January 29, 2015).

## Introduction

The evolutionary trajectory of a protein is guided by structural and functional requirements, resulting in constraints to its amino acid composition and sequence. Thus, functional conservation often results in the conservation of specific sequences. Conversely, multiple amino acid sequences can result in the same three-dimensional (3D) structure, and thus proteins can accept mutations without altering their biological function. This phenomenon is known as protein structure designability, defined as the number of amino acid sequences that have a single structure as their lowest-energy conformation (Emberly et al. 2002). As a result, certain amino acid substitutions are more likely to occur than others in order to maintain a protein’s function and structure. Evolutionary models, or substitution matrices, were developed to describe the probability of one amino acid being replaced by another (reviewed in Thorne 2000). Descriptive capabilities of a substitution model do not exhaust its applications. In the classical phylogenetic analysis pipeline (Anisimova et al. 2013), an appropriate model is essential for most if not all the stages: Identification of homologous sequences, construction of a multiple sequence alignment, and phylogeny inference, which can be followed by more in-depth analyses like inference of sites under selection. General empirical substitution models are mostly based on soluble globular proteins. However, depending on the type of proteins under study, different models are required, describing different constraints and evolutionary trajectories. For example, Brown et al. (2010) show the difference between evolution of proteins with well-defined 3D structure and disordered proteins, lacking well-defined structure and long range interactions, by developing a model for unstructured proteins. Another example is that of proteins encoded by organellar genomes that share different genomic pressures from nuclear ones, prompting Adachi and Hasegawa (1996) and Abascal et al. (2007) to propose models for mitochondrial proteins; similarly Adachi et al. (2000) developed one for chloroplasts. Yet another example is that of transmembrane proteins, where the hydrophobic environment changes both amino acid composition and substitution patterns, requiring thus a specific evolutionary model (Ng et al. 2000). The models mentioned above show improvements, in phylogeny reconstruction and homology detection, over general models for their specific protein classes.

Here, we focus on evolutionary patterns governing the sequence evolution of coiled-coil domains. The coiled-coil is an abundant peptide motif present in all domains of life, which composes up to 10% of all proteins of a species (Liu and Rost 2001). At the sequence level, it is defined by a repetitive heptad pattern (*abcdefg*, (HPPHPPP)_n_) of two hydrophobic amino acids (H, at *ad* positions) separated by two and three polar amino acids (P, at *bcefg* positions). This leads to the emergence of amphiphilic *α*-helices that interact between themselves by their hydrophobic interfaces through interlacing of side chains, known as knob-into-hole packing (Crick 1952), to form a superhelix—the coiled-coil. Coiled-coils were traditionally viewed as rod-like spacers separating functional domains; however, growing evidence suggests that they frequently contain interaction sites and act as protein effectors or scaffolds enabling protein–protein interactions (Zhang et al. 2009; Munro 2011). Proteins containing coiled-coil domains play various biological roles, where the coiled-coil region can act as either (or both) a structural or interacting component. They are involved in transcription regulation (leucine zippers), chromatin and chromosome dynamics (condensins, cohesins); cell cycle; recognition and transport in the endomembrane system (kinesins, dyneins, SNAREs); motility (myosins); structuring organelles (golgins of the Golgi apparatus, Bld10p and SAS-6 of the centrosome, the former was shown [Hiraki et al. 2007] to alter the size and symmetry of the entire organelle when truncated) among many others.

Coiled-coil motifs form well-defined 3D structures that appear in many oligomeric states, yet, they are dominated by simple dimers (Moutevelis and Woolfson 2009; Rackham et al. 2010) that usually form rod-like assemblies, for example, stalks in motor proteins. Although coiled-coils are highly structured, they should substantially differ from globular domains not only in the number of possible folds (secondary structure is restricted just to the *α*-helix) but also in designability: Presence of the heptad pattern limits the sequence space in comparison to an unconstrained *α*-helix. Hence, we expect to observe different evolutionary patterns in coiled-coil domains. However, it is also unclear how conserved coiled-coil sequences are: Is the evolution governed solely by the requirement of the heptad pattern per se, or is the identity of the specific amino acid also of importance? In the first case, we would expect to observe relatively low sequence conservation: Many different amino acid combinations can satisfy the pattern. White and Erickson (2006) showed examples of coiled-coil proteins with different levels of sequence conservation and hypothesized that the conservation depends on the number of interactions along the coiled-coil. They also presented evidence that positions *bcefg* are more constrained in skeletal muscle myosin whereas *ad* positions are more constrained for the analyzed spacer rods. Yet, the general tendency of sequence conservation in coiled-coil regions, compared with globular domains, remains unclear. Here, we address these questions by characterizing the evolutionary patterns of coiled-coil domains and its differences to globular domains. We use this characterization to develop a CC model that shows an improved performance over general models in phylogeny inference and homology detection of coiled-coil proteins.

## Materials and Methods

### Data Sets

Proteomes of all (66) available species were downloaded from the Ensembl database, release 75 (Flicek et al. 2014), which covers Metazoa (largely represented by vertebrates) and *Saccharomyces cerevisiae*. Ensembl Compara was used to retrieve homology information and as a gold set to assess the performance of tested homology prediction methods. Coiled-coil regions were predicted with Paircoil2 (McDonnell et al. 2006) using default parameters. Globular domains were mapped according to the Superfamily database (Gough et al. 2001) using Ensembl’s interface.

### Protein Sequence Alignment

Protein multiple sequence alignments were built with MAFFT, version 7 (Katoh and Standley 2013) with high accuracy mode (––genafpair ––maxiterate 1000).

### Protein Sequence Conservation

A multiple sequence alignment of a protein with its orthologs was used to assess the conservation of amino acids at each position. Conservation was measured using Shannon information entropy $H(X)=-\sum_{i=1}^{n}p(x_{i})\log_{a}\;p(x_{i})$ (Shannon 1948), where *p*(*x_i_*) is the probability (fraction) of the residue *x_i_* in the X column of the alignment. This measures the uncertainty of the given column. Conservation is defined as the difference between the maximum and observed uncertainty, where maximum assumes equal residue probabilities, hence, in general the residue conservation equals:

(1) $$H\prime (X)=\log_{a}\;n+\sum_{i=1}^{n}p(x_{i})\log_{a}\;p(x_{i}),$$

where *n* is the number of symbols in the alphabet (20 for amino acids, 4 for nucleic acids) and *a* usually equals 2 giving bit as the unit of conservation, which leads to a maximum conservation of approximately 4.32 bit for proteins and 2.0 bit for nucleic acids. Columns in an alignment may contain gaps, hence we corrected the conservation value ($H_{c}^{\prime }$) by the fraction of gaps (*f_g_*) in the column $H_{c}^{\prime }(X)=H\prime (X)(1-f_{g})$, for ungapped columns $H_{c}^{\prime }(X)=H\prime (X)$.

### Model Estimation

A set of human proteins containing both coiled-coil regions and globular domains was retrieved and orthologs corresponding to each of these proteins were fetched from Ensembl. Each group of orthologs was aligned to create a multiple sequence alignment. Alignments were restricted to coiled-coil parts by discarding columns containing noncoiled-coil regions. Remaining parts of multiple sequence alignments were inspected for low-quality regions: Any sequence containing greater than 25% gaps, greater than 5% of unknown amino acids (denoted as X) or with average pairwise (the sequence with any other sequence in the alignment) Hamming distance greater than 0.7 were deleted from the alignment. Finally, any column containing greater than 25*%* gaps was also discarded. The total of 2,175 high-quality multiple sequence alignments were used to build the model.

Amino acid substitution rates were estimated using the Expectation Maximization (EM) algorithm (Dempster et al. 1977), implemented in XRate (DART version 0.2, Klosterman et al. 2006), which maximizes the likelihood *L* of a model **Q** given multiple sequence alignments (*D^a^*) and corresponding phylogenetic trees (*T^a^*).

(2) $$L=\prod_{a}L(Q;D^{a},T^{a}).$$

The model was computed using an iterative approach, where the parameter values of the current round are initialized with the parameter values from the previous round, until the likelihood of the model reaches maximum. To initialize the first round of iteration, we tried three models (represented in a form of phylo-grammars; Klosterman et al. 2006): LG (Le and Gascuel 2008), WAG (Whelan and Goldman 2001), and XRate’s nullprot model. Trees were coestimated by XRate based on the input alignments and the initial model: Neighbor-joining followed by EM optimization on the branch lengths (default options). The model was constrained to be reversible (default option). All models converged to similar parameter values and likelihoods. As the final model we chose the one with the highest likelihood—initialized with LG. The model consists of a symmetric amino acid exchangeability matrix **R** and a vector of amino acid equilibrium frequencies **Π**. Assuming a general time reversible model of amino acid substitutions and a constant, independent evolution at each site, **R** and **Π** can be used to create an amino acid substitution matrix **Q**. The relationship between **Q**, **Π**, and **R** is described with the following formulas:

(3) $$\begin{array}{cc} q_{ij} & \;=\pi_{j}r_{ij},\;\;i\neq j\\ q_{ii} & \;=-\sum_{j\neq i}q_{ij}. \end{array}$$

For more information concerning derivation of amino acid substitution models, see Whelan and Goldman (2001) and Le and Gascuel (2008).

The model was then used to derive a series of scoring matrices (**S**) for homology detection, similar to the PAM series (Dayhoff et al. 1978).

(4) $$s_{i,j}=a\;\log_{b}\left(\frac{q_{ij}^{\left(n\right)}}{\pi_{j}}\right),(q_{ij}^{\left(n\right)}\in Q^{n}),$$

where *n* is the PAM distance; $Q^{n}$ denotes matrix exponentiation; *a* and *b* are arbitrary constants (e.g., for PAM250 *a* = *b* = 10). Scores are rounded to the nearest integers.

The entropy of a scoring matrix, average information per residue pair in the alignment, was calculated as follows (Altschul 1991):

(5) $$H=\sum_{i,j}q_{ij}^{*}\;\log_{2}\left(\frac{q_{ij}^{*}}{\pi_{i}\pi_{j}}\right),$$

where $q_{ij}^{*}=\pi_{i}\pi_{j}e^{\ln(2)s_{ij}}$, *s_ij_* is calculated using equation (4) (*a* = 1, *b* = 2), which gives $q_{ij}^{*}=\pi_{i}q_{ij}^{\left(n\right)}$.

### Model Validation

The performance comparison, between the new model and the general one, in phylogeny reconstruction was done using RAxML (Stamatakis 2006). The test set consisted of 179 alignments of orthologous, coiled-coil rich (>25*%*, no globular domain) proteins that were not used for the model estimation. All models included gamma-distributed rate categories, the shape parameter of the distribution was estimated from the data. The F option was used to adapt the model to the empirical amino acid frequencies: The amino acid composition of the multiple sequence alignment. To calculate the difference between obtained estimates, we applied the approach proposed by Le and Gascuel (2008): Measure the Akaike information criterion, AIC (Akaike 1974) for each alignment and use the nonparametric paired sign test on the likelihood values, which are estimated per alignment site, to assess the significance of the difference between models. The average AIC per site is defined as the ratio of the sum of AIC for all alignments given the model and the total number of sites: $\sum_{a}\text{AIC}(M,D^{a})/\sum_{a}s^{a}$. The difference between tree topologies was calculated using the Robinson–Foulds distance (Robinson and Foulds 1981).

### Model Partitioning

For every orthologous group, in the selected subset of coiled-coil proteins, the CC (coiled-coil specific) model was assigned to the alignment region based on the coiled-coil prediction for the human protein, the LG model was used for the remaining part. Phylogenetic analysis was performed with RAxML (Stamatakis 2006). Per site likelihoods of the partitioned method were compared with ones obtained for a single model (either LG or CC) using the Wilcoxon signed-rank test (Wilcoxon 1945).

### Homology Detection

NCBI (National Center for Biotechnology Information) BLAST+ version 2.2.29 (Altschul et al. 1990) was used (with default parameters) for homology prediction. A bidirectional best hit (BBH) algorithm was implemented in a custom Python script. Predictions were validated using Ensembl Compara and Ensembl Pan-taxonomic Compara databases. In order to test the performance of the new model in homology detection, the source code of Basic Local Alignment Search Tool (BLAST) was altered: The CC140 matrix values were included together with the statistical parameters that are used by BLAST with BLOSUM62. The performance of homology detection was analyzed by comparing the values of sensitivity (fraction of actual positives that are correctly identified as such), precision (fraction of positive predictions that are actual positives), and Matthews correlation coefficient (mcc, general performance of a predictor)

(6) $$\text{sensitivity}=\frac{TP}{TP+FN},$$

(7) $$\text{precision}=\frac{TP}{TP+FP},$$

(8) $$\text{mcc}=\frac{TP\times TN-FP\times FN}{\sqrt{\left(TP+FP)(TP+FN)(TN+FP)(TN+FN\right)}},$$

where *TP* is True Positives, correctly identified positives; *TN*, True Negatives, correctly identified negatives; *FP*, False Positives, negatives identified as positives; and *FN*, False Negatives, positives identified as negatives.

## Results

### Sequence Conservation of Coiled-Coils

In order to infer evolutionary relationships between proteins, a certain level of sequence conservation is required. To test whether coiled-coil regions carry phylogenetic information, we measured sequence conservation of coiled-coil regions and compared it with globular domains in a collection of over 2,000 orthologous groups of metazoan proteins. We collected all orthologous groups that have an ortholog in humans, and at least one coiled-coil and one globular domain that serves as internal control. We aligned sequences within each ortholog group and computed the average conservation of corresponding regions: Coiled-coil, globular, and undefined (the remaining part of the protein). We used the Shannon entropy (Shannon 1948) to assess the degree of sequence conservation. The Shannon entropy measures the amount of variation contained at each position in a sequence, which can be interpreted as the level of conservation at that position, and has previously been used, for example by Liu and Bahar (2012) and Schneider and Stephens (1990) in a similar manner. It is suitable to measure conservation in multiple sequence alignments. Conservation is defined as the difference between the maximum possible entropy for a given alphabet (e.g., amino acids) and the observed entropy; hence, conservation of a protein sequence ranges from zero bit (for a random sequence) to approximately 4.32 bit (full conservation).

**Fig. 1.—:**
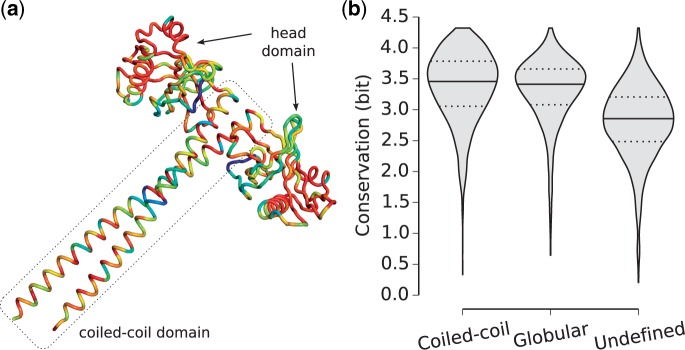
Sequence conservation of protein regions. (*a*) Sequence conservation superimposed on the structure of SAS-6 homolog protein from *Chlamydomonas reinhardtii* (Protein Data Bank: 3Q0X; Kitagawa et al. 2011). Observed conservation ranges from 0.20 to 4.08 bit; blue indicates lowest and red highest conservation. (*b*) Average sequence conservation in human coiled-coil proteins.

As an example, figure 1*a* shows the crystal structure of SAS-6 homolog protein from *Chlamydomonas reinhardtii*, containing both the globular head domain and the coiled-coil tail. Colors represent the sequence conservation at each position between *C. reinhardtii* and multiple metazoan species. On average there is no significant difference between the globular and the coiled-coil parts of this protein, indicating that they contain similar level of phylogenetic information.

Similarly, a high level of conservation among coiled-coil domains emerges from the global analysis (fig. 1*b*). As expected, regions with no domain assignment are less conserved than the ones forming globular domains. In contrast, coiled-coil regions are well conserved; on average, they are even slightly more conserved than globular domains (3.46 bit for coiled-coils and 3.41 bit for globular, median values). The strong conservation of coiled-coil regions is surprising: A certain level of sequence conservation is expected due to the coiled-coil constraint to preserve the heptad pattern, but this result suggests that a specific amino acid sequence is preserved beyond the pattern per se.

Even though the entropy is a measure of sequence conservation, it is not an ideal estimate of phylogenetic informativeness: The rate of evolution of a character at a given time period (Townsend 2007), an indicator of the evolutionary distance between sequences. Yet, a direct estimation of phylogenetic informativeness is more complex; an assumption about the substitution model, the phylogenetic relationship between sequences and intense computation is required (impractical for a large scale analysis). We tested whether entropy can globally approximate phylogenetic informativeness in a comparative analysis on a random sample (200) of sequence alignments. For each alignment, we compared the difference in log-likelihood between the best (as estimated with maximum likelihood) and a random guess of the evolutionary relationship between sequences to assess the amount of phylogenetic information that exists between sequences for alignments build with coiled-coil and globular domains. The observed difference in likelihoods for coiled-coil and globular domains is qualitatively similar to that for entropy (supplementary fig. S1, Supplementary Material online). This suggests that entropy, given its limitations, can roughly approximate global phylogenetic informativeness and is suitable for studies such as this, where a large number of sites and sequences preclude more accurate approaches.

### Substitution Model

In order to study the evolution of coiled-coils, we measured the amino acid frequencies and substitution rates in coiled-coil domains from a collection of over 2,000 orthologous groups of metazoan proteins (see above). After trimming the multiple alignments to remove all noncoiled-coil domains, we developed a substitution model (which we named “CC”) to describe the amino acid exchangeability of the coiled-coil domain, and compared this model with the LG, a general empirical model of protein evolution that was shown to outperform former general models in reconstruction of protein phylogenies (Le and Gascuel 2008).

#### Amino Acid Frequencies

**Fig. 2.—:**
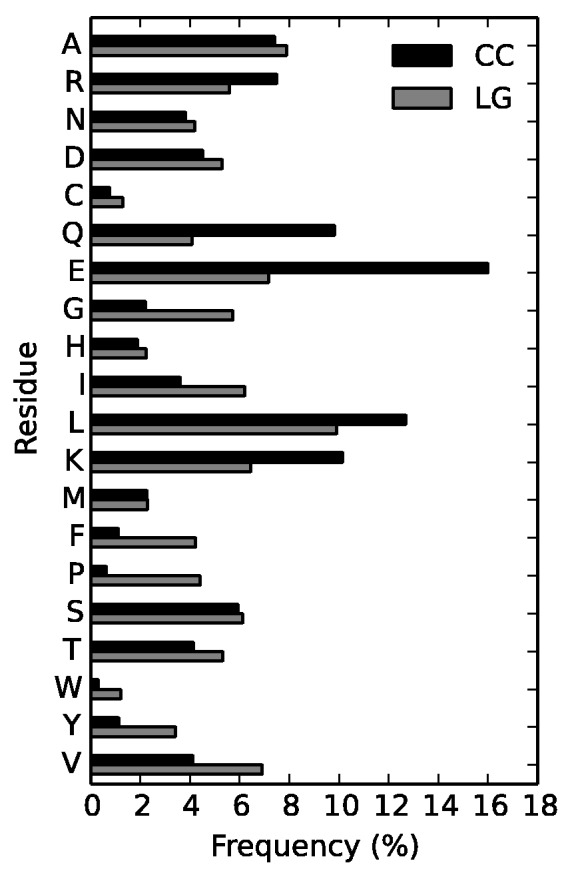
Amino acid equilibrium frequencies (*p_i_*) in CC and LG models.

The amino acid composition of coiled-coil alignments used for creating the CC model (equilibrium frequencies) shows that certain amino acids are preferentially used in coiled-coil regions, whereas others are avoided when compared with globular domains (fig. 2). Charged amino acids with long side chains are more frequent in coiled-coil regions: Negatively charged glutamic acid (E ∼ 16%, the most frequent amino acid), positively charged lysine (K ∼ 11%) and arginine (R ∼ 8%). Glutamine (Q), a neutral, polar amino acid with long side chain, is twice as frequent as in globular domains. Among hydrophobic amino acids leucine (L) is the most common and more frequent compared with the LG model. Aromatic amino acids, that is, tryptophan (W), tyrosine (Y), and phenyloalanine (F) are underrepresented, which is probably due to the exposed nature of the coiled-coil for most of its length to the solvent, whereas globular domains form a hydrophobic core. Similarly, glycine (G), a tiny, flexible amino acid with minimal side-chain (a hydrogen atom) and proline, which disrupts secondary structures, are less common. Our observations are in agreement with the amino acid *α*-helix propensity scale proposed by Pace and Scholtz (1998): EKRQ are more favored in a helix whereas PG are the least favored.

Due to the heptad repeat, (HPPHPPP)_n_, the expected ratio of hydrophobic to polar amino acids in the coiled-coil is 2:5. Unexpectedly, the observed ratio ∼2.5:5 deviates from this ideal case: In some proteins “polar” positions are occupied by hydrophobic residues, which may, for example, lead to the emergence of characteristic, highly stable structures (Deng et al. 2006; Liu et al. 2006). This suggests that evolution of coiled-coils goes beyond maintenance of the heptad repeat. For comparison, in the LG model the ratio is close to 1:1.

#### Amino Acid Substitution Probabilities

**Fig. 3.—:**
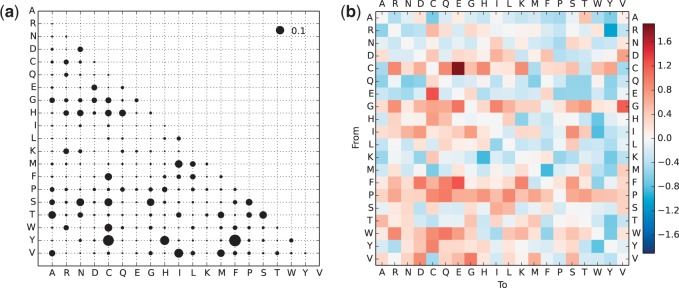
Amino acid exchangeability rates. (*a*) Symmetric matrix of amino acid exchangeability rates for coiled-coil regions in the CC model. The area of each bubble represents the value of exchangeability *r_ij_* between amino acid *i* and *j*. (*b*) Heat map representation of the difference between amino acid substitution rates in CC and LG models. The value for each square is calculated as $\log_{10}\frac{q_{ij}(\text{CC})}{q_{ij}(\text{LG})}$. For both plots, values are scaled so that the expected number of substitutions per site is 1.

Figure 3 shows exchangeability (substitution) rates between amino acids according to the CC model (fig. 3*a*) and the comparison with the general LG model (fig. 3*b*). In the CC model, the frequent amino acids, EQLK (glutamic acid, glutamine, leucine, lysine) have low exchangeability rates, which suggest that they are conserved, with just few exceptions: E↔D (K↔R) where both amino acids are negatively (positively) charged; Q↔H (L↔{F,M}) where Q and H (L and F) are close with respect to the genetic code and substitutions to Q (L) are more frequent than in the opposite direction, due to equilibrium frequencies (see also supplementary fig. S2, Supplementary Material online). In the CC when compared with the LG model, the exchangeability rates for the four frequent amino acids are even lower for most of amino acid pairs, which suggests that EQLK are even more important and less prone to be substituted in coiled-coil regions. Similarly, high exchangeability rates combined with low equilibrium frequencies indicate that proline, tryptophan, and phenylalanine will be preferentially lost in coiled-coils. Glycine is likely to be replaced by alanine (high *α*-helix propensity) or one of the polar amino acids. If we consider long evolutionary distances, some amino acids of similar physicochemical properties will be preferred due to their equilibrium distribution: Glutamic acid (longer side chain, higher propensity to form the *α*-helical structure [Pace and Scholtz 1998]) over aspartic acid, lysine over arginine.

### Phylogenetic Inference with the CC Model

Besides the descriptive capabilities, substitution models are also used for phylogenetic inference. Hence, we assessed the performance of the CC model on phylogeny reconstruction by analyzing 179 orthologous groups of coiled-coil rich proteins (defined as proteins where coiled-coil regions span >25*%* of the sequence and globular domains are absent) and compared the resulting trees with those generated using the LG model, which outperforms previous general models (Le and Gascuel 2008). To insure that we were not artificially improving scores of the CC model over LG, we used different sets of orthologous groups to create the CC model and to compare the performance of phylogeny reconstruction.

We analyzed the overall likelihood of a tree, differences in the tree length and topology. We used the AIC (Akaike 1974) to measure the relative quality of each model for the analyzed data; AIC compares likelihoods of two models taking into account their complexity; hence, a model with more parameters is not necessarily favored over a simpler one. To assess the statistical difference between models, we used the nonparametric paired sign test, similarly to Le and Gascuel (2008). To control for the influence of amino acid equilibrium frequencies on tree estimation, we applied both models together with either the original (model’s) frequencies or frequencies estimated from each of the analyzed alignments (empirical frequencies, +F). Table 1 summarizes the results of the analysis.

**Table 1:** CC and LG Model Comparison with 179 Test Alignments of Coiled-Coil Rich Proteins

M1	M2	ΔAIC (per site)	#${\text{M1}}_{\text{AIC}}{\text{>M2}}_{\text{AIC}}$	#M1 > M2 (*P* < 0.01)	#M2 > M1 (*P* < 0.01)	#T1 > T2 (*P* < 0.01)	#T2 > T1 (*P* < 0.01)
CC	LG	0.57	143	104	23	98	23
CC	LG+F	0.95	154	118	13	113	13
CC+F	LG	0.90	161	145	1	140	1
CC+F	LG+F	1.28	175	148	2	141	2

Note.—Trees were estimated with RAxML under either LG or CC model (+F indicates use of empirical amino acid frequencies), using gamma-distributed rate categories. ΔAIC, average per site difference in AIC between two models (M2 − M1), positive value M1 better than M2. ${\text{M1}}_{\text{AIC}}{\text{>M2}}_{\text{AIC}}$, number of alignments where M1 has a better (lower) AIC value than M2. #M1>M2 (*P* < 0.01), number of alignments where the AIC of M1 is significantly better (lower AIC, P<0.01 for paired sign test on per site likelihood values) than that of M2. #T1>T2 (*P* < 0.01), number of alignments where the AIC of M1 is significantly better than that of M2 and the tree topology differs.

In most cases the CC model produces better trees (lower AIC) than the LG model, even when empirical frequencies are used with LG (LG+F); CC+F model is worse than LG (LG+F) in only 1 (2) case. We obtained similar results when the CC model was compared with two other general empirical models: WAG and JTT (data not shown). The CC model produces shorter trees than LG (∼14% for CC/LG and ∼8% for CC+F/LG+F), indicating that the new model needs to account for fewer hidden substitutions than the general model. Tree topologies obtained with the CC model differ from their LG counterparts for most cases: CC influences the likelihood of the tree, its length and also the shape. We also compared predicted tree topologies with the reference (Ensembl Compara) and observed that the topologies predicted with the CC model are closer to the reference in 42% of the cases, whereas the trees estimated with LG are closer to the reference in 31% of the cases, even though the reference trees are themselves biased toward general models used in the Ensembl pipeline. In 27% of the cases, CC and LG models result in trees that are equally distant to the reference. As a control, we tested the performance of the CC model on globular proteins, and as expected tree likelihoods are worse than for the LG model (data not shown). These results show that the CC model clearly outperforms the general model in phylogeny reconstruction of coiled-coil rich proteins.

### Model Partitioning

Proteins rich in coiled-coil regions but lacking other domains are just a subset of the universe of all coiled-coil proteins. Although it is clear that the CC model is a better choice for reconstructing the phylogeny of coiled-coil rich proteins, selecting an appropriate model for multidomain proteins is more complicated. In those cases model partitioning, assigning different models to specific parts of a protein, should improve phylogenetic inference. We tested whether this is indeed the case on a small set of proteins (that allowed manual inspection of the partitioning scheme) representing different levels of sequence divergence and coiled-coil content (13 proteins compiled in White and Erickson 2006). Model partitioning gives significantly higher tree likelihoods, than either of the models alone, for the majority of tested proteins and is not correlated with the sequence conservation or coiled-coil content (table 2). In five cases we did not observe any significant difference and in only one case a single model gives a better description of the phylogenetic process, indicating that the entire protein evolves according to that model, rather than to two different ones. As a rule of thumb, model partitioning between the CC model and a more general model should lead to better phylogenetic trees. A custom script that assigns a substitution model to the corresponding sequence region based on the coiled-coil prediction and produces an input file for RAxML (Stamatakis 2006) is available at http://www.evocell.org/cgl/resources. An alternative to the manual model selection for a partitioning scheme is to use a semiautomated approach, where the best fitting model is chosen for each predefined partition. This functionality has been implemented in PartitionFinder (Lanfear et al. 2012), yet, the CC model remains to be incorporated into it.

**Table 2:** Model Partitioning in Coiled-Coil Proteins

Protein	Conservation (bit)	Coiled-Coil Content (%)	Best Model
SMC3	3.86	34	—
MYH6	3.78	56	CC+LG
Desmin	3.76	63	CC+LG
KIF5B	3.72	49	CC+LG
SMC1	3.66	46	—
MYH9	3.59	56	CC+LG
SMC4	3.25	39	CC+LG
SMC2	3.09	44	—
KIF4A	3.09	32	CC+LG
Ndc80	3.05	37	—
KIF7	3.03	33	CC+LG
NUF2	2.85	20	CC
NuMA	2.84	67	—

Note.—Phylogenetic inference using a single model or model partitioning in proteins with different sequence divergence and coiled-coil content. The best model is chosen based on the Wilcoxon test, “—” indicates no significant difference between models.

### Homology Detection

Amino acid repeat patterns often present problems for homology detection, by influencing the sequence alignment, which is the common reason to mask low complexity regions. Coiled-coils are based on a relatively simple pattern, hence, it is unclear if the pattern itself is introducing ambiguities in homology detection and deteriorating search performance, an issue raised by several authors (Rose et al. 2004, 2005; Zhang et al. 2009; Rackham et al. 2010; Walshaw et al. 2010; Azimzadeh et al. 2012). We set out to examine to what extent the coiled-coil region influences homology detection, and subsequently test whether the CC model can be used to improve homology detection of coiled-coil proteins.

**Fig. 4.—:**
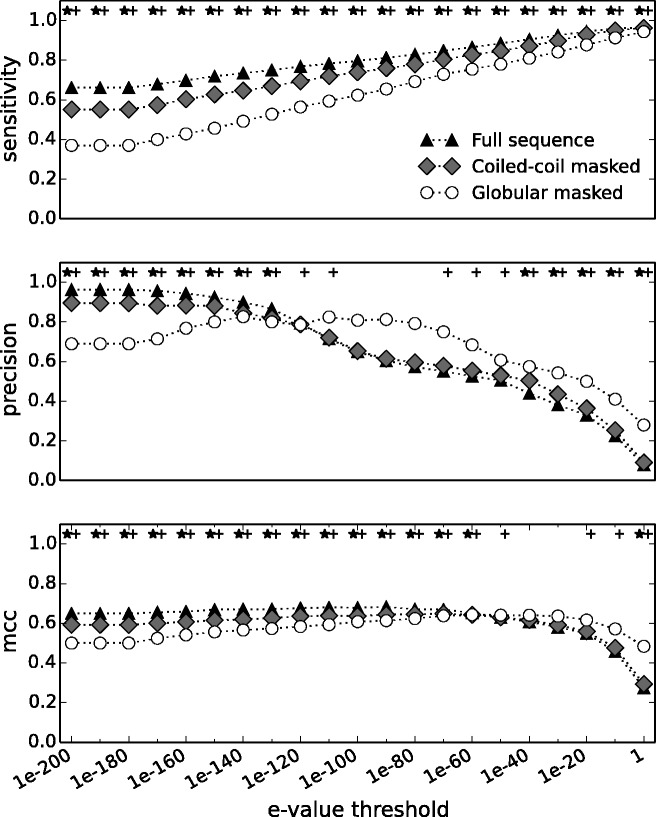
Homology predictions (BLAST) of all human coiled-coil proteins containing at least one globular domain across all species present in the Ensembl database. Sensitivity, precision, and mcc are shown as cumulative plots of median values for each *e* value threshold. ☆+ denotes a significant difference (*P* < 0.01, Mann–Whitney *U* test) between full sequence and masked either coiled-coil regions or globular domains for a given threshold.

To analyze and quantify the influence of the coiled-coil repeat on homology detection we split each sequence into coiled-coil and globular regions (by masking appropriate regions), and used these fragments (as well as the full length sequence for comparison) to detect homologs by performing a search with BLAST (Altschul et al. 1990) against species present in the Ensembl database; predictions were validated based on Ensembl Compara for a range of different *e* value thresholds. We observed that both coiled-coil and globular regions have similar performance (fig. 4). On average the overall sensitivity decreases when the query is restricted to a single domain type, compared with the full sequence query, and the difference is bigger when the coiled-coil is used (globular domain is masked). This effect is more pronounced at very low *e* value thresholds. Interestingly, the change in precision depends on the *e* value threshold; at low thresholds the difference is similar to that of sensitivity, yet, at high thresholds we observed the opposite: Searching with a single domain increases precision and the gain is bigger for the coiled-coil (i.e., when globular is masked). The overall performance (mcc, Mathews correlation coefficient) of homology detection increases when information from both domain types is used, suggesting that the frequent practice of masking coiled-coil domains leads to reduced accuracy when searching for homologs.

Given a query, BLAST searches for similar sequences in a library and assigns a score to putative homologs based on a scoring matrix; the most common matrix used for proteins is BLOSUM62 (Henikoff S and Henikoff JG 1992), the default option in BLAST. Scoring matrices are closely related to substitution matrices: A set of scoring matrices can be derived given a substitution model. The performance of different scoring matrices can be directly compared if the entropy of matrices is similar, even if they were derived using different methods (Altschul 1991).

We decided to test whether using a scoring matrix derived from the CC model can improve homology detection over the standard BLOSUM62 matrix. We created a scoring matrix based on the CC model corresponding to the PAM distance of 140 (Dayhoff et al. 1978), as this has a similar entropy to BLOSUM62, which we will refer to as CC140. A set of CC scoring matrices and a script used to derive them are available at our website (http://www.evocell.org/cgl/resources).

**Fig. 5.—:**
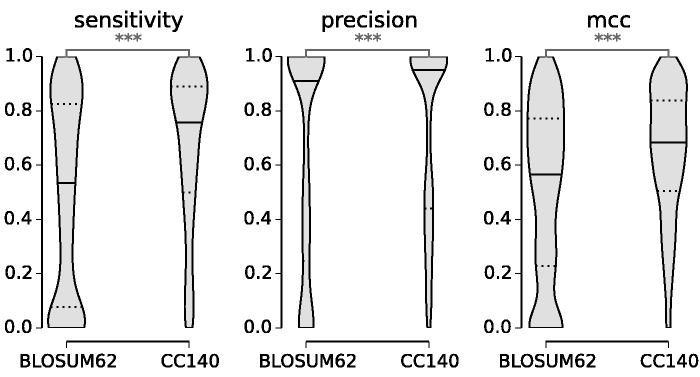
Homology search improvement under the CC model. Homology search comparison between CC140 and BLOSUM62 scoring matrix at the *e* value threshold of 1e-08. Statistical significance between samples was estimated with the Mann–Whitney *U* test (***P<0.001).

To analyze the influence of the scoring matrix on homology detection, we restricted protein queries to coiled-coil regions by masking the remaining part of the sequence and ran a BLAST search with a human sequence as a query against all sequences available in the Ensembl database. In this way, we directly compare the relative performance between matrices on the coiled-coil regions of the sequence. Figure 5 shows the performance comparison between scoring matrices at the *e* value threshold of 1e-08: The CC140 matrix significantly improves both search sensitivity and precision (***P<0.001, Mann–Whitney *U* test). We observed similar gain at lower *e* value thresholds whereas at higher thresholds precision decreases with increase in sensitivity (data not shown). Overall (mmc), the new scoring matrix improves homology detection over BLOSUM62 when used with coiled-coil sequences, irrespectively of the *e* value threshold.

**Fig. 6.—:**
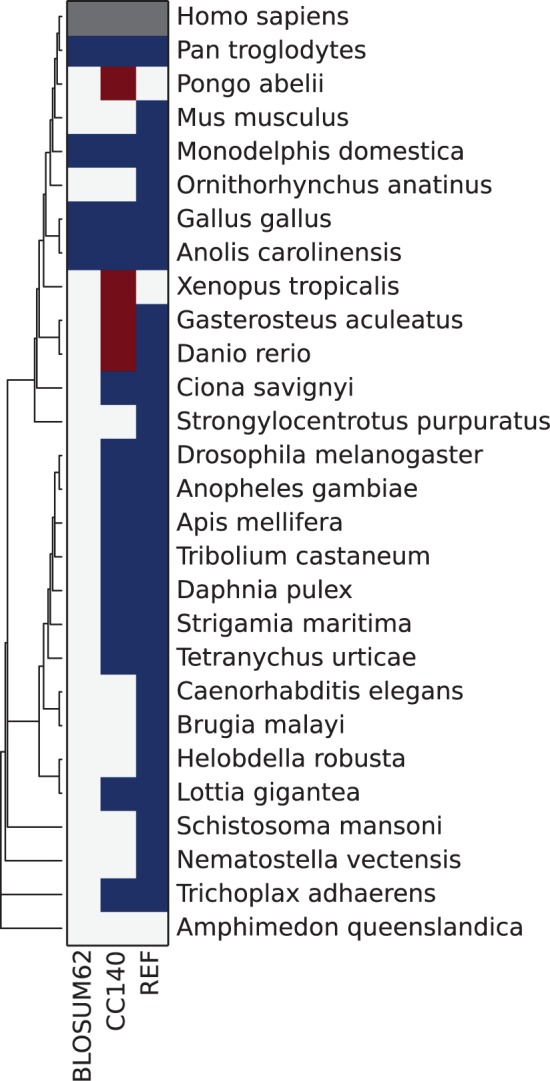
Human PBX4 (ENSP00000251203) orthology prediction against metazoan species with BLOSUM62 and CC140 scoring matrices. Blue/red—correctly/incorrectly assigned ortholog. Ensembl Pan-taxonomic Compara was used as the reference (REF).

We further tested the performance of the new matrix by running orthology prediction with the BBH heuristic (Overbeek et al. 1999) using coiled-coil regions of human proteins against multiple eukaryotic species (present in Ensembl Pan-taxonomic Compara). Similarly to the previous analysis we also observed a significant increase in sensitivity (table 3), albeit of a smaller magnitude. Surprisingly, the biggest difference between matrices occurs within the phylum (Chordata) to which the query species belongs rather than between more distantly related phyla. Subsequently, we tested whether the difference in performance is affected by the length of the coiled-coil query (table 4). Indeed, for short coiled-coil regions (<50 amino acids) the difference is bigger indicating that the new model has relatively higher sensitivity given less signal; however, the gains are still small. Will such small gains be relevant? The following example shows that this is the case. We used BBH for ortholog detection of the human PBX4, where the coiled-coil region spans only 30 amino acids (the remaining part was masked as before). We found that even though CC140 returns four false positives, which in this case are PBX4 paralogs, it overall recovers more true orthologs throughout Metazoa, whereas BLOSUM62 misses all orthologs that belong to more distant groups than reptiles (fig. 6).

**Table 3:** Orthology Prediction Comparison with BLOSUM62 and CC140 Scoring Matrices

	Pan-Taxonomic			Chordata		
	BLOSUM62	CC140	$\Delta_{\text{CC140-BLOSUM62}}$	BLOSUM62	CC140	$\Delta_{\text{CC140-BLOSUM62}}$
Sensitivity (%)	35.42	36.14	0.72***	60.44	62.12	1.68***
Specificity (%)	97.53	97.10	−0.42***	87.25	85.27	−1.98***
Precision (%)	86.39	87.55	1.16	86.80	88.19	1.39*
mcc (%)	39.59	39.94	0.34	24.84	24.59	−0.25

***P<0.001, *P<0.05, Wilcoxon test.

**Table 4:** Orthology Prediction Comparison with BLOSUM62 and CC140 for Coiled-Coil Shorter than 50 Amino Acids

	Pan-Taxonomic			Chordata		
	BLOSUM62	CC140	$\Delta_{\text{CC140-BLOSUM62}}$	BLOSUM62	CC140	$\Delta_{\text{CC140-BLOSUM62}}$
Sensitivity (%)	26.85	27.89	1.05***	47.40	49.88	2.47***
Specificity (%)	97.31	96.55	−0.76***	88.57	86.31	−2.26***
Precision (%)	82.67	85.44	2.77	80.58	84.82	4.24*
mcc (%)	30.87	31.35	0.49	17.68	17.63	−0.05

***P<0.001, *P<0.05, Wilcoxon test.

## Discussion

In this work we described patterns of evolution in coiled-coil sequences, and used these patterns to create a model of evolution that improves phylogeny inference and homology detection of coiled-coils. Despite their repetitive sequence, coiled-coils show a level of sequence conservation similar to that of globular domains. We observed major differences between our model and the general LG model that reflect different properties and constraints of coiled-coil domains, for example, equilibrium frequencies biased to charged and *α*-helix promoting amino acids. We showed that the CC model outperforms general models in phylogeny inference for coiled-coil rich proteins, giving trees with higher likelihoods and often different topologies. Additionally, in the case of multidomain proteins containing both coiled-coil and globular regions, model partitioning is a useful approach to resolve phylogenetic histories, which reflects the fact that distinct folds within a protein may evolve according to different patterns, hence, should be analyzed with different models. Finally, we showed that coiled-coils contain valuable sequence information that can be used in homology detection and that homology detection can be improved by using the CC model.

Our findings are supported by previously reported experimental evidence: Substitutions even between amino acids with similar properties can change the oligomerization state of the coiled-coil. Harbury et al. (1993) demonstrated that by changing hydrophobic residues at *ad* positions in GCN4 leucine zippers, with other hydrophobic residues, two-, three-, and four-helix structures are formed. Similarly, Gonzalez et al. (1996) showed that the Asn16Gln mutation, despite chemical similarity, destabilizes GCN4 allowing two peptide states: Dimer and trimer. Furthermore, Vincent et al. (2013) showed, in a large-scale analysis, that specific pairs of hydrophobic amino acids are more likely to appear in certain oligomeric states. Together those data strongly suggest that although the heptad is necessary for the formation of the coiled-coil structure the specific sequence determines higher orders of organization. We can also expect that the specific sequence may contribute to coiled-coil stability, protein–protein interactions, and possibly other factors.

In order to develop the CC model we used data from Ensembl, a comprehensive database containing sequence information for multiple species and evolutionary relationships between them. The database consists mostly of metazoan species, hence, the model is especially useful to describe the evolution of coiled-coils in animals. Yet, our preliminary findings suggest that this model is also applicable beyond the animal kingdom, and may therefore be a very general model of coiled-coil evolution: 1) We tested the new model on homology detection in plants (supplementary fig. S3, Supplementary Material online) and observed a similar performance improvement over BLOSUM62 to the one seen in animals (fig. 5), and 2) we developed another model, using the same approach, based on protein families containing coiled-coils from Pfam database (Punta et al. 2012), which spans throughout the tree of life and concluded that the model is qualitatively consistent with CC (data not shown). However, in order to correctly define such a broad model we will require a more comprehensive, and qualitatively better, collection of homologous proteins.

An empirical substitution model, such as the one presented here, enables description and interpretation of a protein class by capturing its global biochemical properties. Yet, like all other substitution models, it ignores local patterns within a sequence; future avenues for improvement of the CC model may explore such patterns. One approach could be to implement model partitioning by inferring among-site variation from the alignment, for example, using a mixture model in the context of a Bayesian framework, such as that developed by Lartillot and Philippe (2004) in PhyloBayes, where each site in the alignment falls into one of several classes characterized by its own set of frequencies (CAT model). Although this approach has shown some improvements in phylogenetic inference, especially in the presence of saturation, it is computationally expensive and mostly suited for long alignments due to the necessity of inferring model parameters from the data. Alternatively, in the case of coiled-coils it may be preferable to take advantage of the repetitive nature of the sequence with hidden Markov models, where a hidden state, representing position(s) of the heptad, has an associated phylogenetic model, such as in Thorne et al. (1996) or Goldman et al. (1998). These approaches may bring further improvements in phylogenetic inference and homology detection of coiled-coil proteins.

In this study, we showed that coiled-coils, due to their specific structure and repetitive sequence pattern, differ from globular domains in evolutionary constraints. We used the underlying information contained within coiled-coil regions to develop a new model that both describes evolutionary patterns in coiled-coil sequences and provides an improvement over more general models; one should consider using the CC model to improve the toolkit used in the classical phylogenetic analysis pipeline for coiled-coil proteins.

## Supplementary Material

Supplementary figures S1–S3 are available at *Genome Biology and Evolution* online (http://www.gbe.oxfordjournals.org/).

Supplementary Data
