# Supplementary material for: Evolutionary Patterns in Coiled-Coils
Source: Genome Biol Evol. 2015 Jan 10;7(2):545–56. doi: 10.1093/gbe/evv007 (PMC4350178; doi:10.1093/gbe/evv007)
Supplement: Supplementary Data [file supp_7_2_545__index.html]

Evolutionary Patterns in Coiled-Coils — Supplementary Data 

# Evolutionary Patterns in Coiled-Coils

## Supplementary Data

files

**Files in this Data Supplement:**

- Supplementary Data - pdf file
