# Supplementary material for: Evolutionary Patterns in Coiled-Coils
Source: Genome Biol Evol. 2015 Jan 10;7(2):545–56. doi: 10.1093/gbe/evv007 (PMC4350178; doi:10.1093/gbe/evv007)
Supplement: Supplementary Data [file supp_evv007_evv007-supplementary_materials.pdf]

Supplementary material

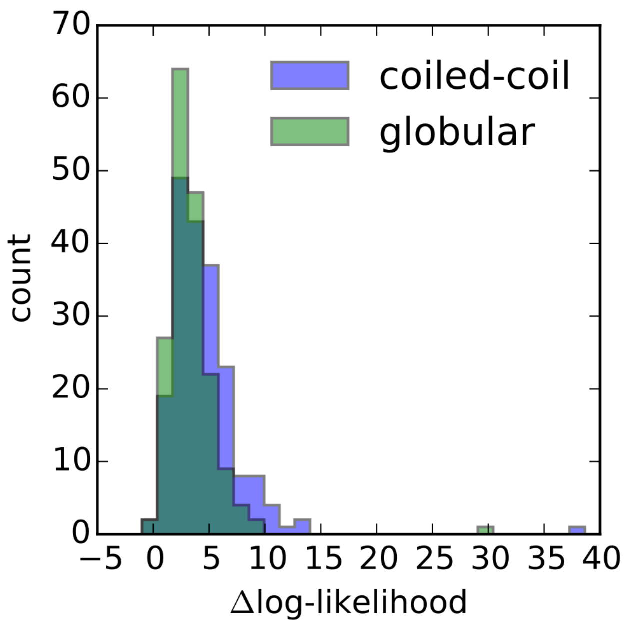

Fig. S1. Per site log-likelihood difference between a random and the best (maximum likelihood) guess of the evolutionary relationship between sequences.

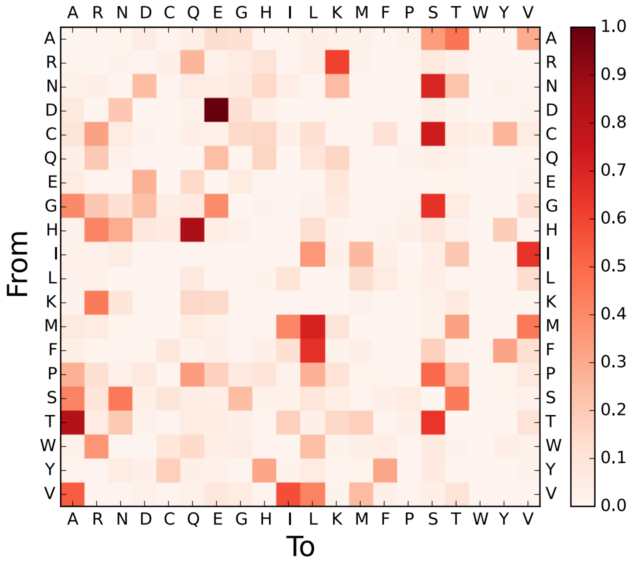

Fig. S2. Amino acid substitution rates (q_{ij}) in the CC model.

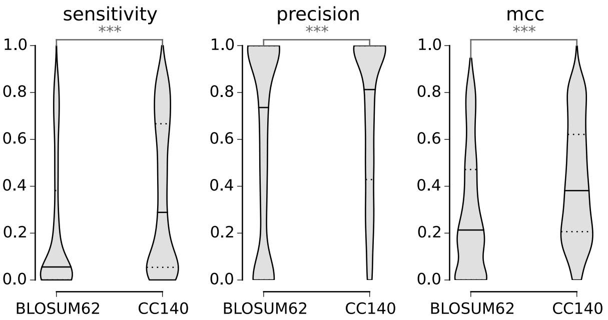

Fig. S3. Homology search improvement under the CC model in plants (Ensembl Plants). Homology search comparison between CC140 and BLOSUM62 scoring matrix at the e-value threshold of 1e-08. Statistical significance between samples was estimated with the Mann-Whitney U test (******* p-value < 0.001).
